# Treatment of disseminated ocular melanoma with sequential fotemustine, interferon α, and interleukin 2

**DOI:** 10.1038/sj.bjc.6600521

**Published:** 2002-10-07

**Authors:** J C Becker, P Terheyden, E Kämpgen, S Wagner, C Neumann, D Schadendorf, A Steinmann, G Wittenberg, W Lieb, E-B Bröcker

**Affiliations:** Department of Dermatology, University of Würzburg, Germany; Department of Dermatology, University of Essen, Germany; Department of Dermatology, University of Göttingen, Germany; Skin Cancer Unit, University Hospital Mannheim, Germany; Department of Dermatology, Friedrich Alexander University of Erlangen-Nürnberg, Germany; Department of Radiology, University of Würzburg, Germany; Department of Ophthalmology, University of Würzburg, Germany

**Keywords:** ocular melanoma, hepatic metastases, extrahepatic metastases, immune therapy, chemotherapy

## Abstract

Malignant melanoma of the uvea is remarkable for purely haematogenous dissemination and its tendency to metastasise to the liver. Although the liver is involved in up to 95% of patients, 50% of these also develop extrahepatic metastases, most often in the lungs, bone, skin, and brain. The only effective treatments reported to date relied on hepatic arterial chemoembolisation or -perfusion. The objective of this study was to establish a therapy protocol addressing patients with both sole liver involvement and systemic disease. Forty-eight patients with metastatic ocular melanoma received fotemustine 100 mg m^−2^ either as 60-min infusion into the hepatic artery or as 15-min infusion via a peripheral vein, depending on the metastatic sites involved, i.e., restriction to the liver or hepatic together with extrahepatic disease. For the first treatment cycle this infusion was repeated after one week. For all cycles, subsequent to a three week resting period, patients received an immunotherapy consisting of subcutaneous interleukin 2 and interferon α_2_. Although objective responses were more frequent within the cohort receiving intraarterial fotemustine (21.7 *vs* 8%), this difference did not translate into a significant benefit in overall survival, i.e., 369 and 349 days, respectively. Of note, this overall survival is much longer than that repeatedly reported for stage IV uveal melanoma not treated with fotemustine, suggesting a therapeutic activity of this cytostatic drug even after systemic administration.

*British Journal of Cancer* (2002) **87**, 840–845. doi:10.1038/sj.bjc.6600521 www.bjcancer.com

© 2002 Cancer Research UK

## 

The annual incidence of uveal melanoma, the second most common type of primary malignant melanoma in humans, is in the range of 0.47 to 0.79 new cases per 100 000 individuals (Shields *et al*, 1996; Chang *et al*, 1998; Woll *et al*, 1999). Improvement of the treatment of primary uveal melanoma over the past years, enabling the preservation of the eye and its remaining visual function, did not, unfortunately, reduce the rate of subsequent tumour dissemination (Shields *et al*, 1996; Seregard, 1996). The 5-year survival rate is approximated between 65 and 81% but decreases to 50% for large tumours (Shields *et al*, 1996).

Uveal melanoma is remarkable for purely haematogenous dissemination and its tendency to metastasise into the liver (Eskelin *et al*, 1999; Gragoudas *et al*, 1991). Hepatic metastases are initially present in 40–60% of patients and, eventually, the liver is involved in up to 95% of patients. Nevertheless, 50% of patients develop extrahepatic metastases, most often in the lungs, bone, skin, and brain. Delayed dissemination is rather frequent; its occurrence, however, restricts life expectancy to less than 5 months (Gragoudas *et al*, 1991).

Metastases are detected by screening examinations or incidentally, with one-third of patients being asymptomatic at the time of diagnosis (Eskelin *et al*, 1999). While annual abdominal ultrasonography and chest X-rays are performed in most European countries for early detection of disseminated disease, some authorities question the value of screening, in the absence of any truly effective therapy for metastatic disease. Indeed, until recently, there had been no effective systemic treatment for metastatic uveal melanoma (Nathan *et al*, 1997). The largest series reported with treatment by DTIC, usually in combination with other drugs and bioactive agents, had a response rate of below 1% with no improvement of survival noted (Bedikian *et al*, 1995; Woll *et al*, 1999).

Regional intraarterial chemotherapy increases the concentration of the antineoplastic drug in hepatic metastases. For uveal melanoma, intrahepatic chemotherapy administration has the theoretical benefit of directly targeting the clinically most relevant site and thereby concentrating the antineoplastic activity. To this end, intraarterial chemoembolisation or perfusion has been reported to induce both complete and partial responses in patients with metastatic uveal melanoma. Bedikian reported a 36% objective response rate induced by cisplatin-based chemoembolisation (Bedikian *et al*, 1995). The group of Leyvraz treated 31 patients by the intraarterial administration of fotemustine, a nitrosurea that is characterised by a high hepatic extraction rate (Leyvraz *et al*, 1997). This treatment resulted in a response rate of 40% with four patients in complete remission (13%). The median overall survival was 14 months with seven patients surviving >2 years. Although the fotemustine concentration in the liver was calculated to be at least eight-fold higher than in normal tissue, the occurrence of myelosuppression with grade III or IV neutropenia or thrombocytopenia was indicative of a systemic spillover. This spillover may explain the low incidence of subsequent extrahepatic disease manifestations despite the long course of the disease, suggesting the systemic activity of fotemustine. Data on systemic fotemustine in uveal melanoma, however, are scarce (Terheyden *et al*, 1998, 2000). If indeed active, systemic fotemustine would be more convenient to administer with the lack of side effects associated with intraarterial hepatic catheters like thrombosis and infection. Its efficacy and toxicity needs thus to be defined in a direct comparison with intraarterial chemotherapy.

At first sight, uveal melanoma seems to be a poor target for immunotherapeutic approaches. They arise in an immune-privileged site that can sustain the growth of foreign tissues due to different mechanisms for suppression of immune responses (Ijland *et al*, 1999; Ksander and Chen, 1999). Notably, lack of expression of MHC class I on primary uveal melanoma was found to be correlated with a better patient survival suggesting natural immunity to exert a protective role in the development of metastatic disease (Blom *et al*, 1997a). Paradoxically, work with animal models for intraocular tumours has demonstrated that adoptively transferred T cells can in principle be effective against such tumours (Sutmuller *et al*, 2000). Moreover, metastases from human uveal melanoma express both MHC class I as well as a variety of melanoma associated antigens, and cell lines established from metastatic lesions are excellent targets for lysis by tumour-specific CTL *in vitro* (Blom *et al*, 1997b; Sutmuller *et al*, 2000). This is in accordance with the fact that several – though still preliminary – clinical reports indicate a therapeutic effect of different immune stimulatory approaches (Dorval *et al*, 1992; Terheyden *et al*, 2000; Woll *et al*, 1999; Nathan *et al*, 1997).

The aim of the present study was to evaluate the activity of a combination of these promising treatment options in a prospective phase II trial. To this end, chemotherapy with fotemustine followed by immune modulation with interleukin 2 and interferon α was given to treat patients with metastatic uveal melanoma. The application route of fotemustine was stipulated according to the presence of sole hepatic tumour manifestations or the occurrence of extrahepatic metastasis.

## PATIENTS AND METHODS

The protocol was in accordance with the Declaration of Helsinki and was approved by the local ethic committees. All patients gave informed consent prior to enrolment into the study.

### Eligibility

Eligibility criteria included measurable sites of distant metastases of uveal melanoma that were not amendable to surgery and did not involve the CNS, evidence of tumour progression in two subsequent staging investigations, and no prior systemic therapy within six weeks of the start of therapy. Each patient was evaluated with a complete history, general physical examination, sonography of the inguinal, axillary and cervical lymph nodes, as well as CT scans of the head, neck, chest, and abdomen. Patients were enrolled into the study and therapy was initiated within 2 weeks after this initial work up. In addition, to be eligible, patients had to have normal blood counts (absolute neutrophil count >1500 μl^−1^ and platelet count >100 000 μl^−1^), liver function (alanine aminotransferase, aspartate aminotransferase, and alkaline phoshatase levels less than two times normal and total bilirubin level <1.5 mg ml^−1^), and renal function (blood urea nitrogen and creatinine levels less than two times normal), a Karnovsky performance score of at least 80%, and a life expectancy of more than three months. Patients with a history or symptoms of a significant cardiac disease were excluded.

### Treatment protocol

**Figure 1:**
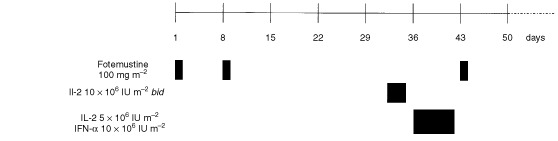
Treatment schedule.

A vignette of the treatment protocol is shown in Figure 1

. Fotemustine (Muphoran®, Servier, France), which is distributed as a freeze-dried sterile powder, was first dissolved in 2 ml ethanol solvent per 100 mg and subsequently diluted in 100 ml 5% dextrose. All these measures as well as the administration to the patient were performed under strict light protection. Fotemustine was applied in a dose of 100 mg m^−2^ either via the hepatic artery over a period of 60 min or via a peripheral vein as a 15-min infusion on day 1. Only for induction, the fotemustine application was repeated on day 8. For the intraarterial infusion, an angiography catheter was placed into the hepatic artery by repeated puncture of the femoral artery. The correct position was controlled by angiography preceding each chemoperfusion. Prophylactic antiemetic treatment consisted of intravenous 5-HT_3_ antagonists such as ondansetron.

The immune modulating therapy was started on day 31 and consisted of subcutaneous injections of 10×10^6^ IU m^−2^ IL-2 *bis in die* on three consecutive days. After a 2 day break, on days 36, 38 and 40 the patient received a subcutaneous dosage of 10×10^6^ IU m^−2^ interferon α_2_ and 5×10^6^ IU m^−2^ IL-2 (3 doses in total). Although the immunotherapy schedule was designed as an outpatient protocol, several patients were hospitalised due to logistic reasons. The following treatment cycles were started with the single dosage of fotemustine 3 days after the last cytokine application (day 43). Vital signs, i.e. blood pressure, heart rate, and temperature, were monitored three times per day. In addition, patients input and output criteria were recorded. Fluid retention of more than 5% of the body weight was treated with furosemide. IL-2 was administered at 50% dose in cases of significant hypotension or when the creatinine level rose to three times of the normal level.

Patients showing evidence of tumour progression after two cycles were regarded as non-responders. Patients with stable diseases or regression after two cycles were offered additional treatment cycles to achieve maximal antitumour activity.

### Evaluation

The first assessment of treatment activity was performed 3 months after initiation of therapy, which was usually prior to the third treatment cycle. Best responses were defined as follows: Complete responses (CRs) characterise the disappearance of all tumour manifestations, whereas partial responses (PRs) indicate decreases of ⩾50% of the sum of the products of all diameters of all measurable lesions; the term objective response (OR) encompassed CR and PR. Stable disease (SD) was defined as a decrease of ⩽50% or an increase of ⩽25% without the appearance of any new lesion. Progressive disease (PD) comprised all stages not already defined, including the appearance of any new lesion. Time to progression, i.e. progression free survival, was measured from the first day of treatment to the first onset of tumour progression. Overall survival was measured from the first day of treatment to the last documented staging examination, i.e. the date last seen. The Kaplan–Meier technique was used to calculate survival data. The log-rank test was used to analyse survival differences among subgroups of patients. All eligible patients were included in the survival analysis which was performed in June 2001.

Bivariate analysis of the relationships between pre-treatment factors and responses were assessed according to the χ^2^, the U-test following Mann–Whitney or the student's *t*-test, depending on the nature and distribution of the data. A *P*-value of <0.05 was considered to be significant. The binominal confidence intervals are given in the range of 95%. All calculations were performed using MEDAS statistical software (Grund EDV-Systeme, Margetshöchheim, Germany).

## RESULTS

### Patients

All patients diagnosed with disseminated ocular melanoma admitted for therapy between December 1996 and October 2000 were considered to be treated according to protocol. Forty-eight patients were found to be eligible to be enrolled into the study. Patients characteristics are listed in Table 1

**Table 1:**
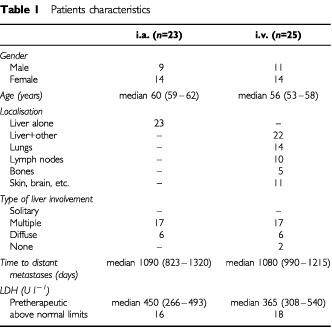
Patients characteristics

. Women accounted for about 60% of all patients. The median time between the diagnosis of the primary tumour and the occurrence of metastases was almost 3 years. Approximately 60% of the eyes had been surgically removed while the others were subjected to brachytherapy. Liver metastases were present in 45 of the patients and in 23 patients this was the only site of metastases; only three patients did not show any sign of liver involvement. If present, liver involvement was generally found in form of multiple or diffuse metastases. Localisation of non-hepatic metastases included the lungs, skin, soft tissues, and bones with decreasing frequency. The majority of the patients were diagnosed with disseminated disease due to symptoms as at that time no formalised follow-up schedule was inaugurated. The median serum lactate dehydrogenase (LDH) level was 450 U l^−1^ for the cohort of patients suffering from liver metastases only and 365 U l^−1^ for those with additional or sole systemic metastases. The upper normal limit for LDH is 240 U l^−1^.

### Toxicity

The most prominent side effect due to fotemustine was thrombocytopenia which occurred in 16 patients but never exceeded grade 3 nor needed thrombocyte substitution (Table 2

**Table 2:**
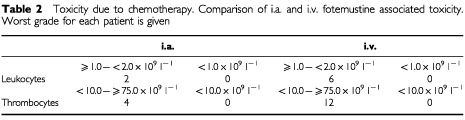
Toxicity due to chemotherapy. Comparison of i.a. and i.v. fotemustine associated toxicity. Worst grade for each patient is given

). A more prominent systemic toxicity of intravenous applications was reflected by the more common occurrence of leukocytopenia in the group of intravenous application of fotemustine. Platelet counts of less 75.0×10^9 ^l^−1^ were observed in 12 patients within the i.v. group in contrast to four patients in the i.a. group (*P*=0.028). It arose most frequently within the first three weeks of the induction cycle. Dose modification of fotemustine due to myelosuppression was only necessary in three patients. Nausea and vomiting was very rare and readily amended by an increased dosage of 5-HT_3_ antagonists. Alopecia was not observed.

In two patients receiving intraarterial fotemustine we observed gastroenteric complications; one patient developed an erosive gastritis, the other a gastric ulcer. These complications are likely due to an atypical blood supply of the stomach via the hepatic artery which was not obvious by angiography. Other complications of the intraarterial catheter included one case of a dissection of the artery wall and one of an angiospasm; both incidences prevented additional courses of intraarterial chemoperfusion. The dissection of the A. hepatica occurred after the fourth intraarterial application of fotemustine in a patient achieving SD. No increase in serum levels of liver enzymes nor alterations of liver function were evident. The angiospasms of the hepatic arteria occurred after two successful i.a. applications during the third attempt. Subsequent cycles were given i.v.

According to immunotherapy, we evaluated 79 treatment cycles in 32 patients. Toxicity was evaluated after two cycles of therapy at the time of the staging using toxicity forms; in patients receiving more than four cycles the subsequent cycles were not documented in such detail (22 cycles). The frequency of toxicities of World Health Organisation (WHO) grade 3 or more are summarized in Table 3

**Table 3:**
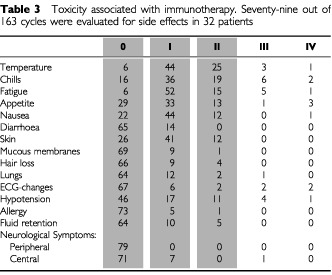
Toxicity associated with immunotherapy. Seventy-nine out of 163 cycles were evaluated for side effects in 32 patients

. Side effects due to immune modulation with IL-2 and interferon α_2_ were regularly present, but rarely severe in nature. No patient was excluded from the study because of unmanageable toxicities. Objective side effects remained stable or decreased with the number of treatment cycles.

### Therapeutic activity

**Figure 2:**
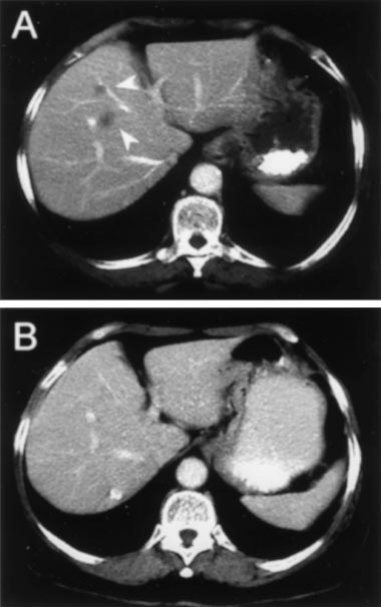
CT scan of the liver depicting metastases prior to therapy (**A**) and the CR after 4 cycles of intravenous fotemustine in combination with subcutaneous IL-2 and IFNα (**B**).

**Figure 3:**
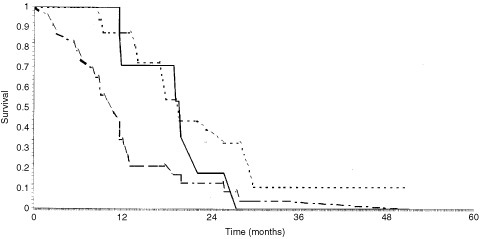
Overall survival according to best clinical response: OR (solid line), SD (dotted line) or PD (dashed line).

Of the 48 patients, only one patient (2%) achieved a CR and six (12.5%) a PR, for an overall response rate of 14.5% (95% confidence interval, 6.1 to 28.4%). Five of these objective responses were observed in the cohort of patients receiving fotemustine intraarterially, while only two (one CR and one PR) of 25 patients in the i.v. therapy group experienced an OR. Figure 2

exemplary depicts resolving liver metastases of the one patient experiencing the CR. The duration of objective responses extended over a median of 450 days, range 273 to 810 days. In addition to these objective responses, we observed that for 18 (37.5%) patients disease progression was stopped or at least slowed down to a degree that they were classified as stable disease. Notably, these patients experienced a median progression free survival of almost one year, range 155 to 855 days resulting in an overall survival similar to that observed after an OR, i.e. the median overall survival of patients with an OR (581 days, range 346 to 826 days) was not significantly improved compared to those with a SD (448 days, range 175 to 1020 days) (Figure 3

). In contrast, the overall survival of patients neither achieving an OR nor a SD was 321 days.

### Prognostic parameters

Based on multivariate analysis only LDH level maintained its role as a pretherapeutic parameter for survival (*P*=0.0058). Other parameters, such as performance status, interval between primary tumour and metastatic disease, sex, age, hepatic function, pretherapeutic serum concentration of S100, or number and localisation of metastatic sites had no significant impact on overall survival.

## DISCUSSION

Despite encouraging new treatment options for disseminated uveal melanoma an effective standard therapy is still missing (Woll *et al*, 1999; Shields, 1993); thus, systematic therapeutic trials are mandatory for the development of an effective treatment regimen. Recent reports demonstrated the prospect of both regional and systemic treatment options (Leyvraz *et al*, 1997; Nathan *et al*, 1997; Salmon *et al*, 1998). However, to date no randomised trial was reported to demonstrate the superiority of either therapeutic strategy. In this respect, the present report is no exception; however, besides being the largest prospective trial in disseminated uveal melanoma reported to date, the obtained data allow to compare the clinical efficacy of the most active single chemotherapeutic drug depending on the administration route, i.e. intraarterial versus intravenous. Although the treatment arms were not randomised, but stratified according to the presence or absence of extrahepatic metastases, the presented data demonstrate the therapeutic activity of systemically applied fotemustine. It is important to note that the patient group receiving intravenous fotemustine should have the worse prognosis as in most patients the liver plus additional sites were involved. Nevertheless, the overall survival for these two groups was comparable with a median of 369 days (range 160 to 1020 days) and 349 days (range 52 to 1535 days), respectively. The higher response rate achieved for the patients in the intraarterial treatment group reflects high response rates for other tumour types (Leyvraz *et al*, 1997). The present study is confirming a previous report by Leyvraz *et al* (1997) on the therapeutic efficacy of fotemustine for the treatment of hepatic metastases of uveal melanoma. However, the incidence of OR induced by fotemustine in that study was significantly higher than the frequency of OR observed by us, i.e. 40 *vs* 22%. Several reasons may explain the advantageous response in the patients population reported by Leyvraz: (1) the majority of patients were subjected to surgical resection of liver metastases, (2) all patients were asymptomatic with respect to the neoplastic disease, (3) the induction cycle consisted of three compared to two dosages of 100 mg m^−2^ fotemustine, and (4) the fotemustine was applied over a period of 4 h compared to 1 h. Notably, in the here presented study all, but two patients with metastatic ocular melanoma seen between December 1996 and October 2000 were considered for treatment according to protocol; thus, a true consecutive series of walk-in patients, not recruited from a screening programme were treated. Nevertheless, the overall survival both for all patients, disregarding the application route, and for those experiencing a benefit from the therapy, i.e. achieving an OR or SD, is very similar in either study ranging around 1 year and 20 months, respectively. As discussed above, a number of experimental and clinical observations suggest a role of the immune system to restrain the progression of uveal melanoma (Ksander and Chen, 1999). In this respect, the surgical resection of only a fraction of the metastatic disease may be detrimental to the patients due to the stress induced immune suppression (Rocha, 1985). The exact contribution of the immune modulation to the therapeutic efficacy of the presented treatment protocol remains to be evaluated. Recent reports on the therapeutic efficacy of IL-2 in combination with histamine in hepatic metastasis of uveal melanoma invigorate the hope that immune modulation may serve as a new tool to treat this disease (Hellstrand *et al*, 2000). Previous attempts to treat metastatic uveal melanoma with chemotherapy relied on regimens established for cutaneous melanoma (Woll *et al*, 1999; Shields, 1993). Despite the fact that these two tumours are derived from the same cell type, many genetic and phenotypic distinctions differentiate them from each other. By means of an *ex vivo* ATP-based tumour chemosensitivity Myatt *et al* (1997) were able to demonstrate that alkylating agents were clearly superior to metabolic inhibitors such as 5-fluorouracil and spindle inhibitors such as vincristine. Moreover, cisplatin or MTIC, i.e. the active metabolite of dacarbazine and temozolomide, showed only a moderate activity in this assay. In their study, the best chemosensitivity of uveal melanoma was observed for the combination of treosulfan and gemcitabine. Unfortunately, fotemustine was not assessed in this study.

The overall 1 year survival rate with metastatic uveal melanoma has been reported at 13%, with a median survival of only 5 months (Woll *et al*, 1999; Kath *et al*, 1993; Shields *et al*, 1996; Pyrhonen, 1998). Subgroup analysis, however, shows a great diversity in overall survival, depending on the presence of different prognostic parameters, such as metastatic sites or elevated LDH. Hence, the clinical outcome of different treatment regimens for metastatic disease is difficult to evaluate if these prognostic parameters are not taken into account (Eskelin *et al*, 1999). Moreover, only a few clinical trials have assessed the impact of systemic therapy and most of these reports were either based on retrospective analysis or small numbers of patients. Thus, metastatic uveal melanoma is still considered to be an incurable disease, with complete responses being only anecdotal (Woll *et al*, 1999; Pyrhonen, 1998). To this end, the most data gained in prospectively designed clinical trials are available for the nitrosurea fotemustine (Leyvraz *et al*, 1997; Salmon *et al*, 1998; Terheyden *et al*, 1998, 2000; Jackel *et al*, 2001).

Fotemustine alkylates the thiolate active sites of three intracellular enzymes, i.e. thioredoxin reductase, glutathione reductase, and ribonucleotide reductase, resulting in their inhibition. Moreover, it has alkylating and carbamoylating effects on nucleic acids (Hayes *et al*, 1997). Both effects may induce the expression of stress molecules such as hsp70 or MICA which can link chemo- and immunotherapy (Weichenthal *et al*, 1998). To this end, fotemustine, in combination with sequential immunotherapy for treatment of metastatic ocular melanoma, resulted in a median survival of one year (95% confidence interval 347 and 390 days); the overall survival of patients experiencing an objective benefit of this therapy in the form of an objective response or a stable disease was 19 months. This survival is substantially better than that reported for other non-fotemustine based treatment options (Woll *et al*, 1999; Pyrhonen, 1998). Since the present study was not prospectively randomised into hepatic arterial *vs* intravenous chemotherapy, the lack of a significant survival benefit of the more invasive therapy can only be taken as a trend advocating such a study.
